# Role of topical antibiotics in prophylaxis against endophthalmitis following intravitreal antibiotics

**DOI:** 10.12669/pjms.345.14817

**Published:** 2018

**Authors:** Asfandyar Asghar, Imran Ellhai, Naila Obaid, Ume Sughra

**Affiliations:** 1 Dr. Asfandyar Asghar, FCPS, FCPS (Vitreo Retina). Department of Ophthalmology, Foundation University Medical College/Fauji Foundation Hospital, Rawalpindi, Pakistan; 2 Dr. Imran Ellhai, FCPS. Department of Ophthalmology, Foundation University Medical College/Fauji Foundation Hospital, Rawalpindi, Pakistan; 3 Dr. Naila Obaid, FCPS. Department of Ophthalmology, Foundation University Medical College/Fauji Foundation Hospital, Rawalpindi, Pakistan; 4 Dr. Ume Sughra, FCPS, MPH. Assistant Professor, Research Associate, Al-Shifa School of Public Health, Al-Shifa Trust Eye Hospital, Rawalpindi, Pakistan

**Keywords:** Intravitreal (IV), Intravitreal bevacizumab (IVB), Post intravitreal endophthalmitis (PIVE)

## Abstract

**Objective:**

To compare the prophylactic use of antibiotic with placebo to prevent post intra-vitreal endophthalmitis and other complications.

**Methods:**

A prospective, cohort study was conducted at Ophthalmology Department, Fauji Foundation Hospital (FFH), from June 2016 to July 2017. A total of 1082 eyes of 673 patients were enrolled in this study. Patients were selected at non-random and divided into two groups. In Group-I, non – exposed, placebo eye drops were given after intravitreal bevacizumab injections and in Group-II, exposed, ofloxacin eye drops were given after intravitreal bevacizumab injections.

**Results:**

Total 1082 eyes were given intravitreal bevacizumab injection in 673 patients. No patients in Group-I, non – exposed, receiving placebo eye drops developed post intra vitreal endophthalmitis, whereas only 01 (0.1%) patient developed post intravitreal endophthalmitis in Group-II, exposed, receiving ofloxacin eye drops. In inferential statistics the P- value was also statistically insignificant [x^2^ (1, N=1082) =0.95, P>0.05]

**Conclusion:**

This study showed that topical antibiotic, as a prophylaxis after intravitreal injections has no role in the prevention of post intravitreal endophthalmitis and other complications.

## INTRODUCTION

Intravitreal (IVT) injections were first introduced in 1911 to introduce air for the treatment of retinal detachment. Later in 1940s, IVT was used to deliver antibiotics for the treatment of endophthalmitis.^1^

At present, many anti VEGF (vascular endothelial growth factor) drugs are used such as pegaptanib, ranibizumab, bevacizumab and aflibercept. While these drugs are beneficial in visual improvement, they have their own complications which has adversely affected the outcome. The continuous use of these injections in treating most of the retinal diseases has increased ocular as well as systemic complications.^2^ Endophthalmitis, though rare, is one of the sight threatening complications of these injections, that has a frequency of occurrence ranging from 0.028% - 1.4%.^3^,^4^

To minimize the risk of endophthalmitis certain prophylactic measures are required. Recent evidence shows that use of preoperative topical povidone iodine is the most effective agent used to minimize the chances of endophthalmitis.^5^ After ocular surgery, topical antibiotics have been the standard of care and this practice has been carried over to intravitreal procedures. However, it is not clear whether these antibiotics have any benefit. Recent literature suggests that topical antibiotics do not reduce the risk of endophthalmitis.^6^,^7^ Rather their repeated usage leads to resistance.^8^

In this study, it remains to be seen how topical antibiotics minimize the post intra vitreal endophthalmitis (IVE) in our own environment. The objective was to compare the prophylactic use of antibiotic with placebo to prevent post intra-vitreal endophthalmitis and other complications.

## METHODS

A prospective, cohort study was conducted at Ophthalmology Department, Fauji Foundation Hospital (FFH), from June 2016 to July 2017. Ethical committee of hospital has approved this study. Total 1082 eyes of 673 patients were included. Age of the patients ranged from 25-85 years and both genders were included. Sampling technique was non-probability convenience sampling. Patients diagnosed with proliferative diabetic retinopathy (PDR), diabetic maculopathy (DM), Diabetic vitreous haemorrhage, non-ischemic central retinal vein occlusion (CRVO) and wet type of age related macular degeneration (AMD) were included. Patients having stye, blepharitis, acute conjunctivitis, scleritis, raised IOP (intraocular pressure) greater than 30 mmHg and raised blood sugar level (greater than 210 mg/dl) on the day of intravitreal injection and patient who were lost to follow up were excluded.

Detailed eye examination was performed in all patients. This included visual acuity, anterior segment examination with slit lamp biomicroscope, and intraocular pressure (IOP) checked with Goldmann Applanation Tonometer. After pupillary dilatation, fundus examination was carried out with 90 dioptre (D) lens. Peripheral retinal evaluation was performed using indirect ophthalmoscope and, where indicated, ultrasonography (B- scan) was performed. In FFH, we planned to give intravitreal bevacizumab (IVB) to our patients on three consecutive days every month. 15-20 patients were booked for each day.

On each day, ophthalmologist and assistant, before making IVB, scrubbed with manorapid (antiseptica), wore head cap, mask, sterilized gown and gloves. On first day after removal of plastic seal from the vial, keeping aluminum seal and rubber seal intact drew 1-2 ml of bevacizumab with the help of 3cc disposable syringe depending upon the number of patients included on that particular day. The needle of the syringe was then replaced by new sterilized 23 gauge (G) needle. One cc insulin syringe with 29 G needle was taken and, after removal of piston of insulin syringe, 0.05 ml (1.25 mg) bevacizumab was injected from behind without touching the tip of insulin syringe. 10-15 injections of bevacizumab 0.05 ml (1.25 mg) were prepared. The plastic seal of vial of bevacizumab was again placed over the top with sticking placed over it. Then this vial was stored at four degree centigrade in a refrigerator. On second and third day of IVB injection, we took out vial of bevacizumab from refrigerator, and removed the plastic seal of the vial. The rubber seal of the vial was doused with 10% povidone iodine for 3-4 minutes, which was wiped out with 70% alcohol swab. Again required numbers of injections were prepared in same manner for each patient under aseptic conditions. Consent of the patient was obtained and blood sugar was checked before each intravitreal injection. In all patients before intravitreal injection, 10% povidone - iodine was also used to clean the eyelids and orbital adenexa. Proparacaine (Alcain) was then instilled 2- 3 times with an interval of 4-5 minutes, followed by 5% povidone – iodine instillation in conjunctival sac region for 2-3 minutes before IVB. Sterilized towel and speculum was used in each case. IVB was given 3.5 mm or 4.00 mm away from limbus depending upon the status of lens. Each patient was given a dose of 1.25 mg in 0.05 ml, in minor operation theatre. After IVB injection, 5% povidone - iodine was again instilled in conjunctival sac in both groups. IOP was checked with the help of air puff tonometer 30 minutes after intravitreal injection. Patients were divided into two groups based on non- randomized technique. In Group-I, non-exposed, placebo artificial tear eye drops were given after IVB injection and in Group-II, exposed, ofloxacin eye drops were given after IVB injections.

Follow up of the patients was after 24 hours, 07 days and 30 days to see any signs of endophthalmitis or any other complications. Data was collected using a structured preset proforma. Frequencies and percentages are shown for qualitative variables, mean and standard deviations for quantitative variables. Data was analyzed using SPSS version 17

## RESULTS

It was a cohort study design; a total 1082 eyes of 673 patients were included. Patients were divided into two groups. Group-I, non – exposed, placebo included 513 eyes (329 patients). Group-II, exposed, ofloxacin included 569 eyes (344 patients). In Group-I, 41 patients and in Group-II, 49 patients did not come for follow up and were excluded from the study. Females’ predominance was seen in both groups because in FFH, families of ex-servicemen are entitled for treatment. In Group-I, mean age was 60.30± 8.8 years and in Group-II, mean age was 54.4±9.6 years. Descriptive analyses are shown in Table-I. DM was the most common disease found for which IVB was given in both groups, as shown in Table-II. In Group-II which was exposed and were given ofloxacin eye drops, developed post IVE after IVB. No patient in Group-I which was non-exposed and was given placebo, developed post IVE as shown in Table-III. After putting data into 2X2 table the incidence of complications were calculated for both exposed and non exposed groups by dividing the number of complications by the total number of exposed 10.5/100 eyes and non exposed 8.77/100 eyes respectively. The relative risk is 1.1 which showed no difference in the rate of complications among exposed and non- exposed population of patients, as shown in Table-IV.

**Table-I:** Gender of responds (N Pt = 673).

	Group - I (N Pt=329	Group - II (N Pt=344)
1. Male	16 (4.8%)	16 (4.6%)
2. Female	313 (94.8%)	328 (95.1%)

Number = N, Patient = Pt.

**Table-II:** Diagnosis of patients included in the study (N Pt=673).

	GROUP - I (N Pt=329)	GROUP - II (N Pt=344)
1. Diabetic maculopathy	205 (62.1%)	222 (64.3%)
2. PDR	37 (11.2%)	36 (10.4%)
3. Wet ARMD	13 (3.9%)	09 (2.6%)
4. CRVO	36 (10.9%)	42 (12.2%)
5. BRVO	20 (6.1%)	23 (6.7%)
6. Diabetic vitreous haemorrhage	18 (5.5%)	12 (3.5%)

PDR = Proliferative diabetic retinopathy, ARMD = Age related macular degeneration, CRVO= Central retinal vein occlusion, BRVO = Branch retinal vein occlusion.

**Table-III:** Rate of complications following intravitreal injections (N EYES=1082).

S No.		Group-I (N Eye = 513)	Group-II (N Eye=569)	Chi-Square X^2^	P-Value
1.	Subconjunctival haemorrhage	21(4.09%)	35(6.1%)		
2.	Glaucoma	13(2.5%)	17(2.9%)		
3.	Corneal abrasion	04(0.7%)	04(0.7%)		
4.	Congestion at injection site	07(1.3%)	03(0.5%)		
5.	Endophthalmitis	00	01(0.1%)		
	Total	45	60	0.95	> 0.05

**Table-IV:** 2X2 TABLE for the calculation of incidence rates and relative risk.

		Complication	No Complication	Total N Eyes
1. Group-II (exposed to Ofloxin)	60	509	569
2. Group-I (non-exposed, Artificial Tear)	45	468	513
3. Total N Eyes	105	977	1082

Incidence of complications among exposed Group-II= 60/569X 100 = 10.54/100 patients Incidence of complications among non-exposed Group-I= 45/513X 100 = 8.77/100 patients RELATIVE RISK (RR) = [INLINE] = 10.54/ 8.77 =1.12

**Fig.1:**
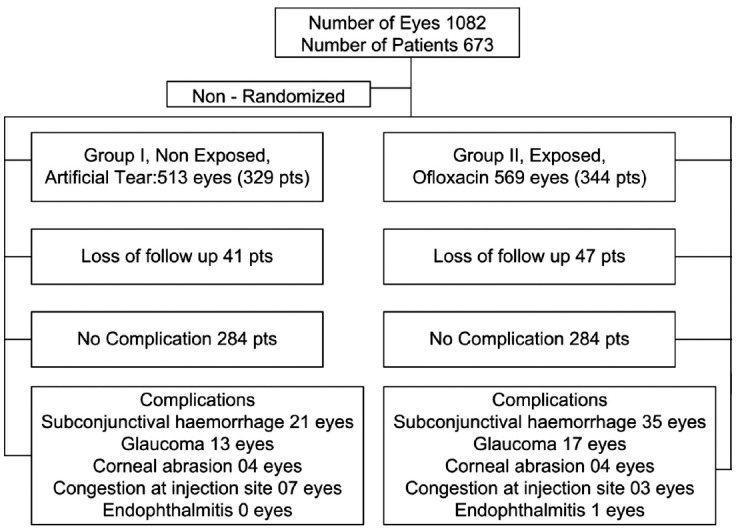
Cohort Study design of the research and the distribution of participants in groups.

## DISCUSSION

The incidence of endophthalmitis found in this study was 0.1%. Reported incidence of endophthalmitis ranges from 0.019% to 1.6%.^2^,^9^ Findings of this study correlate with international literature. In this study, staphylococcus aureus was the organism found. International studies showed Streptococcus organism is the most common organism found in intravitreal injections as compared to other intraocular surgery.^2^ The difference in causative organisms in these two settings has been attributed to the contamination of injection field by aerosolization or droplet spread.^2^ A reason of streptococcus organism was that most of the intravitreal injection was given without facemask. In this study, all the surgeons used to wear facemask while giving intravitreal injections. This might be one of the reasons why streptococcus was not causative agent.

The most important aspect that an ophthalmologist should consider while giving intravitreal injections is the selection of patients and precautions taken during injection. Active external infection, including significant blepharitis, must be treated before the use of injection.^9^ In this study, we excluded all those patients who were suffering from active external infection. The use of 5% povidone– iodine in the conjunctival fornices is an accepted universal practice and is a strong recommendation for preventing endophthalmitis.^10^ We used 5% povidone– iodine before and after IVB injections in both groups. This might be one of the reasons why endophthalmitis incidence was quite low in our study. It has become a universal trend to use post injection topical antibiotics assuming that their use reduces the risk of infection; however, there is evidence disputing this assumption.^7^ Moxifloxacin, ofloxacin and trimethoprim/polymyxin-B antibiotics are used for the treatment of ocular surface infections. However, none has shown reductions in the incidence of post procedure endophthalmitis.^11^ On the other hand, Apurva G et al concluded, in their review of literature, statistically significant (p = 0.029) decrease in the rate of endophthalmitis when moxifloxacin was used (0.01%) as compared to antibiotics (0.04%) like ciprofloxacin, ofloxacin, gatifloxacin, polymyxin and tobramycin.^12^ Recently, Kessel L et al in a meta-analysis, found no evidence that topical fluoroquinolone antibiotics can prevent endophthamitis.^13^ In this study; we have found that even if ofloxacin eye drops were given to patients, endophthalmitis was still diagnosed.

The use of topical antibiotics, pre and post operatively in cataract surgery, is the norm followed around the world. In this intraocular procedure patients are given antibiotics may be once in his life. In contrast, patients with wet exudative AMD receive topical antibiotics for years on regular intervals after each intravitreal injection.^4^,^14^ Thus repeated use of antibiotics has the potential to develop resistant bacteria strains.^11^ That is why fluoroquinolone resistance is an emerging problem in ocular microbiology.^15^-^18^

### Limitations of the study

It has small sample size, single centered and culture sensitivity test was not performed to prove that patient developed resistance to antibiotics. This is an ongoing study and collaboration with other centers will enable us to have better understanding and control of infections after intravitreal injections.

Strength of this study is that it is prospective and comparative in nature. It is probably the first study of its kind in Pakistan showing that topical antibiotics have no role in decreasing the chance of post IVE.

## CONCLUSION

This study showed that topical antibiotic, as a prophylaxis after intravitreal injections has no role in the prevention of post IVE.

### Authors’ Contribution

**AA:** Conceived, designed.

**IE, NO:** Data collection and manuscript writing.

**US:** Data collection, Statistical analysis & editing.
